# Commentary: Does the Norwegian Police Force Need a Well-Functioning Combat Mindset?

**DOI:** 10.3389/fpsyg.2020.572324

**Published:** 2020-11-30

**Authors:** Swen Koerner, Mario S. Staller

**Affiliations:** ^1^Department of Training Pedagogy and Martial Research, German Sport University Cologne, Cologne, Germany; ^2^University of Applied Sciences for Police and Public Administration, Aachen, Germany

**Keywords:** police education and training, critical incidents, decision making, combat mindset, ecological dynamics, police operation

Boe et al. (2020) have recently raised the remarkable question of whether the Norwegian police (and the police in general, p. 2) is in need of a well-functioning combat mindset. In the light of the series of ongoing current incidents and public debates regarding police work on an international level, the issue tackled by the authors is socially and scientifically of high relevance and raises a number of follow-up questions, last but not the least essentially challenging the present organization of police education and training in Norway. In view of their former studies primarily on military performance in demanding operations, Boe et al. (2020) explain “that a combat mindset is of great import (sic!) when it comes to coping with complex and unpredictable situations” (p. 2). To illustrate the relevance of such a combat mindset, the authors refer to the terrorist events that shook Norway on the 22nd of July 2011 and caused the country to suffer the largest terrorist attack since World War II. As Boe et al. (2020) assume, police performance before, during, and after the terrorist attacks lacked a *combat mindset*, defined as “willingness and ability to continue the fight despite high levels of mental and physical pain” (p. 2), which leads them to the conclusion “that the Norwegian Police Force could certainly have spent more time and resources on developing a better combat mindset” (p. 3). Although we fully appreciate that the authors aim at critically reflecting upon police operation to optimize further the preparation of police officers, we raise several concerns about how the authors arrive at the conclusion of their article. The following commentary will cover five major aspects that we feel warrants additional mention and may extend and differentiate some of the suggestions.

In view of an ecological dynamics approach (Araújo et al., 2006), police operations address demanding tasks in dynamic, sometimes unforeseen situations and environments. The performance here can be viewed as the product of a dynamic interaction of individual, collective, task, and environmental constraints (Körner and Staller, 2018), requiring (a) the optimal and effective interlocking of mental, emotional, and physical components at the individual level, (b) of interpersonal components at the squad level, and (c) in social coordination with other squads and operational units in larger critical incidents. Against this background, the assumption of an allegedly missing individual combat mindset for the alleged failure of the Norwegian police equals a noteworthy reduction of complexity. Although Boe et al. (2020) themselves express uncertainties regarding this causal attribution, the assumption forms the argumentative core of their article.

The causal explanation is also reductionist in methodological terms: it is carried out without data related to the targeted component of an individual officer's mindset. Instead, Boe et al. (2020) refer to “own experiences of educating and training police and military officers” (p. 3), not considering alternative explanations.

As performance and expertise are task-specific and context-dependent (Gobet, 2016), neither individual statements made by an *American* police officer about police training in the USA (p. 5) nor statements made by a Norwegian *military* officer with regard to his mission in Afghanistan (p. 4) substantiate the validity of the combat mindset or a change in curriculum for the Norwegian police in a sufficient manner. The authors do not further problematize the change of domain and national context carried out here or potential effects on police (see p. 5); on the contrary, the statements serve as justification for their demand for a combat mindset of Norwegian police officers.

This leads to a constant feature of the argumentation. Boe et al. (2020) repeatedly refer to older sources and, more importantly, to work derived from popular combat culture (e.g., Siddle, 1995; Lichtenfeld and Yanilov, 2001; Murray, 2004; Grossman and Christensen, 2008), which lacks scientific rigorousness. As a result, the discussion of supposed components of the combat mindset falls behind the current state of research and evidence. Thus, for example, the concept of decision making presented is based on a traditional rationale that is discussed based on literature from 1933 (Von Schell, 1933). Underlying assumptions of these decision-making models and differences to current conceptualizations of decision-making (Roycroft and Roach, 2019) and their (potential) effects on behavior in critical situations are neither mentioned nor discussed.

Finally, although Boe et al. (2020) concede *not* having founded their evaluation of the events on “detailed information” (p. 2), they nonetheless conclude that the Norwegian Police Force lacked a well-functioning combat mindset. The reverse conclusion performed by the authors that (in case less combat mindset has been the problem) more combat mindset is the solution has to be confronted with arguments of the ongoing debate on guardian vs. warrior mindset (Stoughton, 2014, 2016; McLean et al., 2019), the reliance on military tools, strategies, and tactics within the police domain (Mummolo, 2018) and research on the relationship between personality attributes and job performance (Aamodt, 2004; Cuttler and Muchinsky, 2006; Varela et al., 2004). For example, the concept of the warrior mindset has been met with criticism, particularly Stoughton (2014). A narrow focus on enhancing skills for combat may come at the expense of developing the skills needed for non-violent conflict resolution (Staller and Körner, 2020). Recent evidence showed (Wolfe et al., 2020) that a broader perspective on conflict may reduce the use of force. Potential risks of using military educational strategies and the potential development of a mindset that perceives the world as an inherently dangerous place are neither mentioned nor discussed. This is in line with the reasoning of the cited popular police combat culture (Grossman, 1995; Asken et al., 2010); however, empirical evidence suggests a connection between perceiving the world as a dangerous place and the engagement in violence (Huesmann, 2018). Finally, the demand for a combat mindset has to be questioned against the body of evidence, indicating the relevance of personal determinants such as conscientiousness, emotional stability, and agreeableness for functional police performance.

We strongly agree with Boe et al. (2020) that the education and training of police officers must be strictly geared to the requirements of the performance context. However, these requirements are versatile in nature and include an array of social interaction options with different dynamics. Therefore, training of police officers has to be context-specific; this implies that research of and for the police, along with the drawn conclusion, also acknowledges this context-specificity.

To summarize, the article by Boe et al. (2020) indicates a reduction of complexity and generalization and selective use of research and data. The concept of a well-functioning combat mindset introduced as a causal explanation for the incident on July, 22nd 2011 in Norway is neither empirically evident nor in a strict sense plausible; therefore, its explanatory value that is heavily claimed by the authors remains vague. More importantly, the concept itself, along with its components like decision making, needs to be discussed along with current concepts, arguments, and empirical evidence available.

## Author Contributions

SK and MS authors contributed equally to the ideas presented. SK wrote the first draft of the paper. Both authors contributed equally to editing the first draft to its final version.

## Conflict of Interest

The authors declare that the research was conducted in the absence of any commercial or financial relationships that could be construed as a potential conflict of interest.
